# Surveillance of the rabies-related lyssavirus, Mokola in non-volant small mammals in South Africa

**DOI:** 10.4102/ojvr.v88i1.1911

**Published:** 2021-08-03

**Authors:** William C. McMahon, Jessica Coertse, Teresa Kearney, Mark Keith, Lourens H. Swanepoel, Wanda Markotter

**Affiliations:** 1 Centre for Viral Zoonoses, Department of Medical Virology, Faculty of Health Sciences, University of Pretoria, Pretoria, South Africa; 2 Centre for Emerging Zoonotic and Parasitic Diseases, National Institute of Communicable Diseases, National Health Laboratory Services, Johannesburg, South Africa; 3 Ditsong National Museum of Natural History, Pretoria, South Africa; 4 Mammal Research Institute, Department of Zoology and Entomology, Faculty of Natural and Agricultural Sciences, University of Pretoria, Pretoria, South Africa; 5 Department of Zoology, University of Venda, Thohoyandou, South Africa

**Keywords:** Bushveld gerbil, lyssavirus, Mokola, non-volant small mammal, rabies-related, reservoir, rodent, surveillance

## Abstract

The reservoir host of Mokola virus (MOKV), a rabies-related lyssavirus species endemic to Africa, remains unknown. Only sporadic cases of MOKV have been reported since its first discovery in the late 1960s, which subsequently gave rise to various reservoir host hypotheses. One particular hypothesis focusing on non-volant small mammals (e.g. shrews, sengis and rodents) is buttressed by previous MOKV isolations from shrews (*Crocidura* sp.) and a single rodent (*Lophuromys sikapusi*). Although these cases were only once-off detections, it provided evidence of the first known lyssavirus species has an association with non-volant small mammals. To investigate further, retrospective surveillance was conducted in 575 small mammals collected from South Africa. Nucleic acid surveillance using a pan-lyssavirus quantitative real-time reverse transcription polymerase chain reaction (qRT-PCR) assay of 329 brain samples did not detect any lyssavirus ribonucleic acid (RNA). Serological surveillance using a micro-neutralisation test of 246 serum samples identified 36 serum samples that were positive for the presence of MOKV neutralising antibodies (VNAs). These serum samples were all collected from *Gerbilliscus leucogaster* (Bushveld gerbils) rodents from Meletse in Limpopo province (South Africa). Mokola virus infections in Limpopo province have never been reported before, and the high MOKV seropositivity of 87.80% in these gerbils may indicate a potential rodent reservoir.

The Mokola virus (MOKV), a rabies-related lyssavirus, represents one of 17 recognised species within the *Lyssavirus* genus, all capable of causing a fatal encephalitic disease (Walker et al. 2018). The Mokola virus is exclusively endemic in Africa with only 30 sporadic cases reported since its discovery more than 50 years ago (Figure 1; Table 1) (Coertse et al. 2017; Kgaladi et al. 2013). The reservoir host of MOKV is still unknown, with spillover dead-end hosts such as domestic cats (*Felis catus*) and dogs (*Canis familiaris*), most commonly reported to be infected with MOKV. This has led to the hypothesis that the reservoir of MOKV might be a prey species that interacts with domesticated animals via a prey-to-predator pathway (Kgaladi et al. 2013). Non-volant small mammals (i.e. shrews, sengis and rodents) have been suggested as possible reservoir hosts considering that previous MOKV isolations were in shrews (*Crocidura spp.*), four in Nigeria and one in Cameroon (Causey et al. 1969; Kemp et al. 1972; Le Gonidec et al. 1978), and a single reported case in a rodent (*Lophuromys sikapusi*) in the Central African Republic (Saluzzo et al. 1984). To investigate further, nucleic acid and serological surveillance were retrospectively conducted, targeting non-volant small mammals from specific locations in South Africa.

**FIGURE 1:**
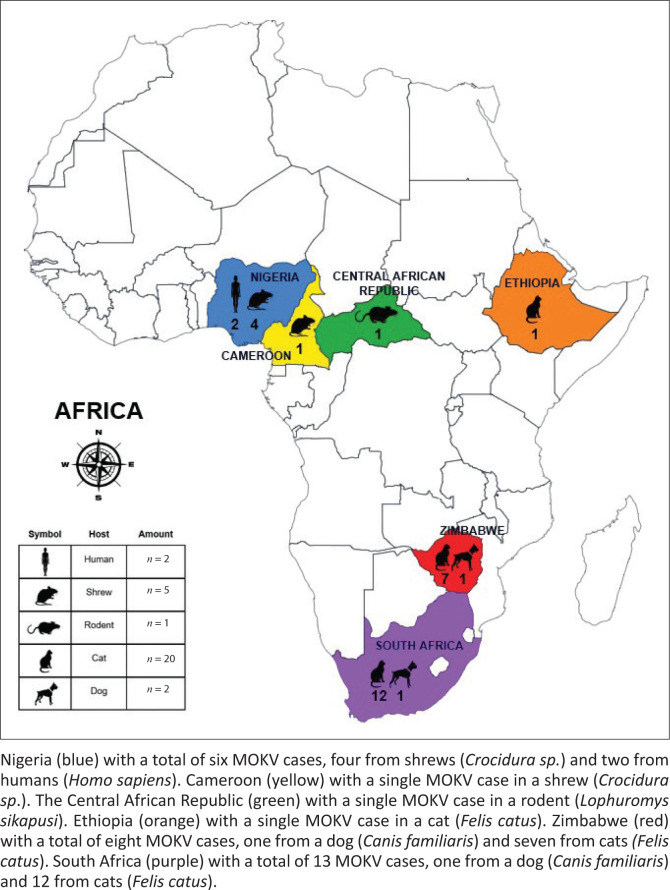
Geographical distribution of all reported Mokola virus cases (*n* = 30) in the African continent.

**TABLE 1:** Summary of all reported Mokola virus cases in Africa.

Date	Virus/Laboratory Reference Numbers	Host Species	Detection Material	Geographical Location	Reference‡
**Nigeria (*n* = 6)**					
May 1968	IbAn 26801†	*Crocidura sp.* (Shrew)	Organ pool (heart, lung, liver, spleen & kidney)	Ife Farm, Ibadan, Nigeria	Causey and Kemp (1968); Kemp et al. (1972)
May 1968	IbAn 27157†	*Crocidura sp.* (Shrew)	Organ pool (heart, lung, liver, spleen & kidney)	Private residence, University of Ibadan, Ibadan, Nigeria	Causey and Kemp (1968); Kemp et al. (1972)
July 1968	IbAn 27377† RV4	*Crocidura sp.* (Shrew)	Organ pool (heart, lung, liver, spleen & kidney)	Mokola, Ibadan, Nigeria	Causey and Kemp (1968); Kemp et al. (1972)
August 1968	IbAn 29777†	*Homo sapiens* (Human)	Cerebrospinal fluid (CSF)	Inalende, Ibadan, Nigeria	Familusi and Moore (1972); Kemp et al. (1972)
December 1969	IbAn 51715†	*Crocidura sp.* (Shrew)	Organ pool (liver & spleen)	Virus Research Laboratory, Ibadan, Nigeria	Causey and Kemp (1969); Kemp et al. (1972)
March 1971	IbAn 56909†	*Homo sapiens* (Human)	Brain	Idikan, Ibadan, Nigeria	Familusi and Moore (1972); Kemp et al. (1972)
**Cameroon (*n* = 1)**					
January 1974	An Y1307† RV39 86100CAM	*Crocidura sp.* (Shrew)	Organ pool (brain, liver & spleen)	Nkol-Owona, Yaounde, Cameroon	Le Gonidec et al. (1978)
**Central African Republic (*n* = 1)**					
October 1981	AnRB3247† RV40 86101RCA	*Lophuromys sikapusi* (Rodent)	Brain	Botami, Bangui, Central African Republic	Saluzzo et al. (1984)
**Ethiopia (*n* = 1)**					
1989–1990	Eth-16† RA 133/82 RV610	*Felis catus* (Cat)	Brain	Addis Ababa, Ethiopia	Mebatsion, Cox and Frost (1992)
**Zimbabwe (*n* = 8)**					
April 1981	12017†	*Felis catus* (Cat)	Brain	Bulawayo, Zimbabwe	Foggin (1982); Foggin (1988)
May 1981	12245†	*Felis catus* (Cat)	Brain	Bulawayo, Zimbabwe	Foggin (1982); Foggin (1988)
June 1981	12341†	*Felis catus* (Cat)	Brain	Bulawayo, Zimbabwe	Foggin (1982); Foggin (1988)
August 1981	12574†	*Felis catus* (Cat)	Brain	Bulawayo, Zimbabwe	Foggin (1982); Foggin (1988)
October 1981	12800†	*Canis familiaris* (Dog)	Brain	Bulawayo, Zimbabwe	Foggin (1982); Foggin (1988)
March 1982	13270†	*Felis catus* (Cat)	Brain	Bulawayo, Zimbabwe	Foggin (1983); Foggin (1988)
April 1982	13371† Zim82 RV1035	*Felis catus* (Cat)	Brain	Bulawayo, Zimbabwe	Foggin (1983); Foggin (1988)
November 1993	21846† RV1017	*Felis catus* (Cat)	Brain	Selous, Zimbabwe	Bingham et al. (2001)
**South Africa (*n* = 13)**					
December 1970	700/70† V21.G3 V241	*Felis catus* (Cat)	Brain	Umhlanga Rocks, KwaZulu-Natal, South Africa	Meredith and Nel (1996); Nel et al. (2000)
July 1995	543/95†	*Felis catus* (Cat)	Brain	Mdantsane, Eastern Cape, South Africa	Meredith and Nel (1996); Nel et al. (2000)
February 1996	112/96† RV1021	*Felis catus* (Cat)	Brain	East London, Eastern Cape, South Africa	Von Teichman et al. (1998); Nel et al. (2000)
May 1996	322/96†	*Felis catus* (Cat)	Brain	Yellow Sands, Eastern Cape, South Africa	Von Teichman et al. (1998); Nel et al. (2000)
May 1997	252/97† V552.S3	*Felis catus* (Cat)	Brain	Pinetown, KwaZulu-Natal, South Africa	Von Teichman et al. (1998); Nel et al. (2000)
May 1997	229/97† V550.S3	*Felis catus* (Cat)	Brain	Pinetown, KwaZulu-Natal, South Africa	Von Teichman et al. (1998); Nel et al. (2000)
March 1998	071/98† V635.S3 RA361	*Felis catus* (Cat)	Brain	Pietermaritzburg, KwaZulu-Natal, South Africa	Von Teichman et al. (1998); Nel et al. (2000
June 2005	404/05†	*Canis familiaris* (Dog)	Brain	Nkomazi, Mpumalanga, South Africa	Sabeta et al. (2007)
March 2006	173/06†	*Felis catus* (Cat)	Brain	Farm near East London, Eastern Cape, South Africa	Sabeta et al. (2007)
2008	226/08†	Felis catus (Cat)	Brain	Grahamstown, Eastern Cape, South Africa	Sabeta et al. (2010)
June 2012	12/458†	*Felis catus* (Cat)	Brain	Durban, KwaZulu-Natal, South Africa	Coertse et al. (2017)
July 2012	12/604†	Felis catus (Cat)	Brain	Durban, KwaZulu-Natal, South Africa	Coertse et al. (2017)
January 2014	14/024†	Felis catus (Cat)	Brain	Pietermaritzburg, KwaZulu-Natal, South Africa	Coertse et al. (2017)

Sp., species; RV, rabies virus; IbAn, Ibadan.

† , The original virus reference number as indicated in the reference article(s);

‡ , References form part of Appendix 2.

Non-volant small mammals were captured and sampled in accordance with the field procedure guidelines of Sikes and Gannon (2011) during the period of 2015–2017 from two different sites in South Africa: Meletse area in Limpopo province (24.5914° S, 27.6258° E) and Secunda area in Mpumalanga Province (26.5158° S, 29.1914° E). All the species investigated were designated as of Least Concern by The International Union for Conservation of Nature Red List of Threatened Species. Morphological species identification followed classifications by Meester et al. (1986), Newbery (1999), as well as Monadjem et al. (2015). Following morphological identification, animals were anesthetised with Isofor (Safeline Pharmaceuticals, South Africa), after which blood was collected by cardiac puncture (1% – 3% volume/body mass) in 0.8 mL MiniCollect serum separator tubes (Greiner Bio-One, Austria). Serum was separated from whole blood by centrifugation (Centrifuge 5418, Eppendorf, Germany) at 4300 g for 5 min and transferred to 2.0 mL Sarstedt tubes (Sarstedt Inc.). Animals that were not collected as voucher specimens were marked with a unique tattoo number near the base of their tail, and released back to their respective capture sites. Voucher specimens were euthanised with an overdose of Isofor, after which their organs were harvested (i.e. brain, tongue, salivary glands, heart, kidney, lungs, pectoral muscle, spleen, intestines, rectum and bladder) in 2.0 mL Sarstedt tubes for a broader pathogen surveillance study and immediately stored in liquid nitrogen until storage at –80 °C. Carcasses were placed in a 3 L PathoPak (Intelsius Solutions, United Kingdom [UK]) containing 80% ethanol and were submitted to Ditsong National Museum of Natural History and the Natural History Collection for Public Health and Economics for voucher-based morphological identification, and museum archiving.

Total ribonucleic acids (RNAs) were extracted from brain samples (*n* = 329) (nine shrews, four sengis and 316 rodents) using TRIzol™ reagent (Invitrogen, United States [US]), followed by nucleic acid surveillance using a pan-lyssavirus quantitative real-time reverse transcription polymerase chain reaction (qRT-PCR) assay as previously described (Coertse et al. 2019). Serum samples (*n* = 246) (three shrews, four sengis and 239 rodents) were subjected to serological surveillance using a micro-neutralisation test as previously described (Smith & Gilbert 2017), during which MOKV 12/458 (2012, *Felis catus*, Durban, KwaZulu-Natal, South Africa) (Coertse et al. 2017) was used as challenge virus. If a reduction or absence of fluorescence was observed at the 1:25 serum dilution during initial screening, the serum sample was subjected to follow-up screening (in duplicate) at 1:10, 1:50, 1:250 and 1:1250 serum dilutions. The 50% end-point (ED) neutralisation titre was calculated by the Reed and Muench method (1938) and considered positive for Mokola virus neutralising antibodies (MOKV VNAs) when they had a 50% ED neutralisation titre at a serum dilution of ≥ 25 (i.e. where ≤ 5 out of the 10 counted fields contain infected cells at the 1:25 serum dilution). If additional material was available, non-volant small mammals that tested positive for the presence of MOKV VNAs were subjected to genetic species identification with the Cytochrome B (CytB) barcoding PCR assay as previously described (Greenberg et al. 2012). Template deoxyribonucleic acid (DNA) required for the barcoding assay was extracted from various biological sample types (such as blood, kidney, heart and pectoral tissue) using the Quick-DNA™ Miniprep Plus Kit (Zymo Research, US).

All of the brain samples were negative for the presence of viral RNA with the pan-lyssavirus qRT-PCR assay (Appendix Table 1-A1). Negative results were expected as these animals were apparently healthy individuals and did not exhibit any visible signs of disease. An overall MOKV seropositivity of 87.80% (36 out of 41) was observed for the gerbils (*Gerbilliscus leucogaster*) tested from Meletse at the cut-off 1:25 serum dilution (Figure 2; Appendix Tables 1-A1, 2-A1, 3-A1 & 4-A1). The titre ranges for this rodent species were high when compared to another serological surveillance study conducted in Zimbabwe (Foggin 1988). Foggin identified MOKV VNAs in 5.63% (18 out of 320) of all rodents that were tested. An overall MOKV seropositivity of 17.57% (13 out of 74) was observed for gerbils which neutralised MOKV infection at various serum dilutions that ranged from 1:8, 1:16 to 1:32. None of the other MOKV serological surveillance studies have tested this rodent species for the presence of MOKV VNAs (Aghomo et al. 1990; Kemp et al. 1972; Nottidge, Omobowale & Oladiran 2007; Ogunkoya et al. 1990). Even though MOKV has been shown to cross-react in serological assays with other closely-related lyssaviruses (Kuzmin et al. 2008), cross-reactivity with other phylogroup II lyssaviruses was not investigated in this study.

**FIGURE 2:**
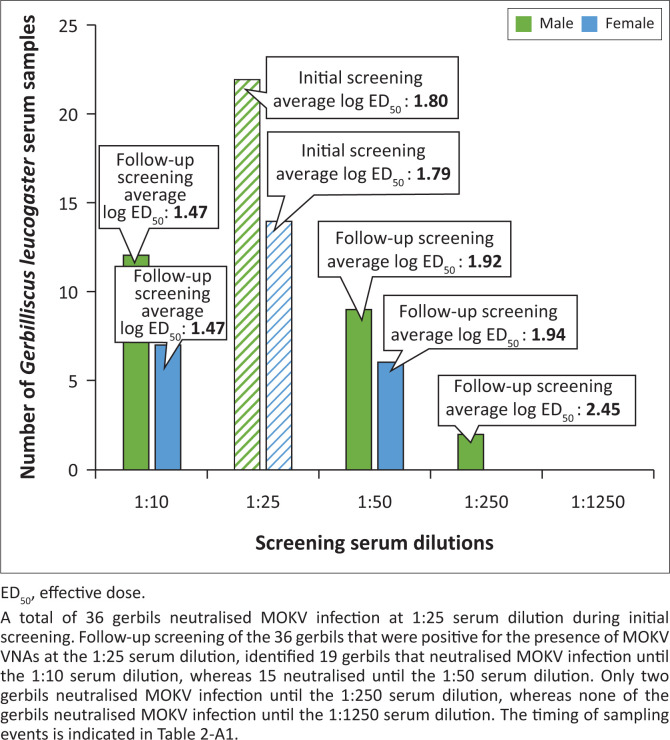
Graphical representation of the micro-neutralisation test results of the *Gerbilliscus leucogaster* serum samples from Meletse (*n* = 36).

Of the 36 gerbils showing MOKV seropositivity, only 28 were genetically identified with the CytB barcoding PCR assay (Table 2). The same identification was obtained from morphological examination of 24 voucher specimens (Table 2). Eight gerbils could not be identified to species level as they were released and no additional sample material was available. The Highveld gerbil, *Gerbilliscus brantsii,* is sympatric with *G. leucogaster*, however, based on known museum records, no *G. brantsii* has been caught at Meletse before (Rautenbach 1982) and these were, therefore, allocated to *G. cf. leucogaster*. The variability observed in the per cent identity (i.e. 83.78% – 100.00%) between the individual gerbils is expected since previous molecular characterisation assays performed on the *Gerbilliscus* genus have recorded intra-species genetic variation that range from 1% to 20% (Aghová et al. 2017; Colangelo et al. 2007).

**TABLE 2:** Genetic and morphological species identification and voucher information for all *Gerbilliscus leucogaster* serum samples from Meletse, Limpopo province that were positive for the presence of Mokola virus neutralising antibodies.

UP reference number	Sample information	Museum information†		Genetic identification information‡					
	Original morphological identification (Field)	Museum number	Morphological identification confirmation	PCR assay	DNA source	Query cover (%)	Per cent (%) identity	GenBank accession number§	Genetic identification (BLAST result)
UP4962	*Gerbilliscus sp.*	TM49197	*Gerbilliscus leucogaster*	CytB	Pectoral	99	97.78	AJ875295	*Gerbilliscus leucogaster*
UP12086	*Gerbilliscus sp.*	TM49248	*Gerbilliscus leucogaster*	CytB	Kidney	100	99.79	AJ875294	*Gerbilliscus leucogaster*
UP12133	*Gerbilliscus sp.*	TM49251	*Gerbilliscus leucogaster*	CytB	Kidney	66	84.33	AJ875295	*Gerbilliscus leucogaster*
UP12166	*Gerbilliscus leucogaster*	TM49259	*Gerbilliscus leucogaster*	CytB	Heart	98	94.85	AJ875295	*Gerbilliscus leucogaster*
UP12183	*Gerbilliscus leucogaster*	N/A	*Gerbilliscus leucogaster*	-	-	-	-	-	-
UP12185	*Gerbilliscus leucogaster*	TM50540	*Gerbilliscus leucogaster*	CytB	Rectum	86	98.31	AJ875294	*Gerbilliscus leucogaster*
UP12187	*Gerbilliscus leucogaster*	TM50541	*Gerbilliscus leucogaster*	CytB	Pectoral	100	99.57	AJ875294	*Gerbilliscus leucogaster*
UP12193	*Gerbilliscus leucogaster*	TM50542	*Gerbilliscus leucogaster*	CytB	Lung	97	93.33	AJ875295	*Gerbilliscus leucogaster*
UP12194	*Gerbilliscus leucogaster*	NHCPHE_MAM-20	*Gerbilliscus leucogaster*	CytB	Kidney	100	99.60	KM454057	*Gerbilliscus leucogaster*
UP12195	*Gerbilliscus leucogaster*	NHCPHE_MAM-21	*Gerbilliscus leucogaster*	CytB	Kidney	100	95.10	AJ875295	*Gerbilliscus leucogaster*
UP12196	*Gerbilliscus leucogaster*	N/A	*Gerbilliscus leucogaster*	CytB	Blood	100	89.29	AJ875295	*Gerbilliscus leucogaster*
UP12197	*Gerbilliscus leucogaster*	NHCPHE_MAM-22	*Gerbilliscus leucogaster*	CytB	Kidney	100	96.59	KM454057	*Gerbilliscus leucogaster*
UP12202	*Gerbilliscus leucogaster*	NHCPHE_MAM-23	*Gerbilliscus leucogaster*	CytB	Kidney	96	99.16	AJ875294	*Gerbilliscus leucogaster*
UP12207	*Gerbilliscus leucogaster*	TM50543	*Gerbilliscus leucogaster*	CytB	Lung	100	93.40	KM453987	*Gerbilliscus leucogaster*
UP12208	*Gerbilliscus sp.*	NHCPHE_MAM-3	*Gerbilliscus leucogaster*	CytB	Kidney	99	86.76	KM453992	*Gerbilliscus leucogaster*
UP12221	*Gerbilliscus sp.*	N/A	*Gerbilliscus leucogaster*	CytB	Blood	100	99.57	AJ875294	*Gerbilliscus leucogaster*
UP12223	*Gerbilliscus sp.*	TM50544	*Gerbilliscus leucogaster*	CytB	Pectoral	100	97.23	AJ875295	*Gerbilliscus leucogaster*
UP12246	*Gerbilliscus leucogaster*	TM50545	*Gerbilliscus leucogaster*	CytB	Pectoral	100	97.87	AJ875295	*Gerbilliscus leucogaster*
UP12259	*Gerbilliscus sp.*	TM50546	*Gerbilliscus leucogaster*	CytB	Pectoral	100	100.00	AJ875294	*Gerbilliscus leucogaster*
UP12296	*Gerbilliscus leucogaster*	NHCPHE_MAM-24	*Gerbilliscus leucogaster*	CytB	Heart	66	83.78	AJ865294	*Gerbilliscus leucogaster*
UP12297	*Gerbilliscus sp.*	NHCPHE_MAM-5	*Gerbilliscus leucogaster*	CytB	Kidney	100	90.71	KM453986	*Gerbilliscus leucogaster*
UP12303	*Gerbilliscus leucogaster*	TM50547	*Gerbilliscus leucogaster*	CytB	Pectoral	100	99.15	AJ875294	*Gerbilliscus leucogaster*
UP12307	*Gerbilliscus leucogaster*	TM50548	*Gerbilliscus leucogaster*	CytB	Kidney	98	85.41	KM453992	*Gerbilliscus leucogaster*
UP12350	*Gerbilliscus leucogaster*	N/A	*Gerbilliscus leucogaster*	-	-	-	-	-	-
UP12354	*Gerbilliscus leucogaster*	N/A	*Gerbilliscus leucogaster*	-	-	-	-	-	-
UP12373	*Gerbilliscus leucogaster*	N/A	*Gerbilliscus leucogaster*	CytB	Blood	100	94.03	KM453987	*Gerbilliscus leucogaster*
UP12426	*Gerbilliscus sp.*	N/A	*Gerbilliscus leucogaster*	CytB	Blood	100	94.24	KM454060	*Gerbilliscus leucogaster*
UP12431	*Gerbilliscus sp.*	N/A	*Gerbilliscus leucogaster*	-	-	-	-	-	-
UP12457	*Gerbilliscus sp.*	N/A	*Gerbilliscus leucogaster*	-	-	-	-	-	-
UP12517	*Gerbilliscus sp.*	NHCPHE_MAM-25	*Gerbilliscus leucogaster*	CytB	Pectoral	100	99.57	AJ875294	*Gerbilliscus leucogaster*
UP12518	*Gerbilliscus sp.*	NHCPHE_MAM-26	*Gerbilliscus leucogaster*	CytB	Kidney	96	97.66	AJ875295	*Gerbilliscus leucogaster*
UP12524	*Gerbilliscus sp.*	TM50549	*Gerbilliscus leucogaster*	CytB	Heart	81	97.10	AJ875294	*Gerbilliscus leucogaster*
UP12526	*Gerbilliscus sp.*	TM50550	*Gerbilliscus leucogaster*	CytB	Kidney	97	97.45	AJ875295	*Gerbilliscus leucogaster*
UP12539	*Gerbilliscus sp.*	N/A	*Gerbilliscus leucogaster*	-	-	-	-	-	-
UP12543	*Gerbilliscus sp.*	N/A	*Gerbilliscus leucogaster*	-	-	-	-	-	-
UP12553	*Gerbilliscus sp.*	N/A	*Gerbilliscus leucogaster*	-	-	-	-	-	-

UP, University of Pretoria; DNA, deoxyribonucleic acid; N/A, not available; TM, Transvaal museum; NHCPHE_MAM, Natural History Collection of Public Health and Economics; CytB, Cytochrome B; PCR, polymerase chain reaction; BLAST, Basic Local Alignment Search tool.

† , Museum information (i.e. museum number & morphological identification) for vouchers in Ditsong National Museum of Natural History (TM) and Natural History Collection of Public Health and Economics (NHCPHE_MAM), N/A refers to not available because no voucher was taken;

‡ , Genetic identification information: (1) PCR assay refers to the molecular barcoding assay that was used to determine the genetic identity of the rodent – Cytochrome B (CytB); (2) DNA source refers to the material that was used to extract DNA from for the PCR assay; (3) Query cover refers to how much the submitted sequence (i.e. the query sequence) is covered by the target sequence; (4) Per cent identity refers to the similarity of the query sequence to the target sequence; (5) GenBank accession number refers to GenBank’s reference for the target sequence; (6) BLAST results refer to the genetic identity (i.e. genus and species name) of the target sequence’s organism;

§ , The genus and species names associated with the listed GenBank accession numbers from the BLAST results refer to *Tatera leucogaster. T. leucogaster* underwent a taxonomic name change in 2005 and is currently referred to as *Gerbilliscus leucogaster*.

Members of the *Gerbilliscus* genus are nocturnal and terrestrial, exhibit no sexual dimorphism (Skinner & Chimimba 2005) and occupy simple to complex, deep burrows (i.e. warrens) (De Graaff 1981; Granjon & Dempster 2013). They are physiologically, morphologically and behaviourally adapted to live in arid climates (Granjon & Dempster 2013; Monadjem et al. 2015). *Gerbilliscus leucogaster*, however, is less arid adapted and can be found along rivers and drainage lines in open grasslands and wooded savannas (Dempster 2013; Monadjem et al. 2015). The breeding pattern and social organisation of *G. leucogaster* rodents are not well-understood, however, studies have reported a communal nature (De Graaff 1981; Smithers 1971) with burrows being occupied by a pair (Skinner & Chimimba 2005) and some warrens housing families or several adults (Choate 1972). The ecological nature of Bushveld gerbils may potentially be the reason why this specific rodent species are more likely to be MOKV seropositive compared to solitary rodent species belonging to the *Steatomys* and *Rhabdomys* genera occurring at Meletse.

More nucleic acid and serological surveillance studies in non-volant small mammal populations are required to obtain a better understanding of MOKV distribution, prevalence and its potential reservoir species. Brain and serum samples in this study were collected from seemingly healthy small mammals in areas that do not coincide with areas where previous MOKV cases have been reported in South Africa. Surveillance should be expanded to areas where MOKV spillover infections in cats and dogs have previously been reported. Furthermore, because lyssavirus distribution and dynamics might be influenced by seasonality, surveillance efforts should also include samples that were collected in different seasons and over multiple years. This expansion, together with representative sample sizes of certain non-volant small mammal species, will collectively increase the possibility of identifying more of these animals that are infected or that have previously been exposed to MOKV.

## Appendix 1

**TABLE 1-A1:** Non-volant small mammal species included in the surveillance of Mokola virus in South Africa.

Non-volant small mammal type	Non-volant small mammal species	Brain samples		Serum samples	
		Amount tested	Amount positive	Amount tested	Amount positive
**Meletse, Limpopo, South Africa (*n* = 473)**					
Shrews (*n* = 9)	*Crocidura hirta*	2	0	2	0
	*Crocidura maquassiensis*	1	0	1	0
	*Suncus lixus*	3	0	0	0
Sengis (*n* = 8)	*Elephantulus brachyrhynchus*	4	0	4	0
Rodents (*n* = 457)	*Acomys selousi*	2	0	3	0
	*Aethomys* sp.	1	0	16	0
	*Aethomys ineptus*	30	0	23	0
	*Aethomys chrysophilus*	20	0	16	0
	*Gerbilliscus* sp.	1	0	5	5
	*Gerbilliscus leucogaster*	33	0	36	** 31**
	*Graphiurus murinus*	3	0	2	0
	*Lemniscomys rosalia*	9	0	7	0
	*Mastomys coucha*	45	0	23	0
	*Mastomys natalensis s.l.*	13	0	12	0
	*Micaelamys* sp.	0	0	3	0
	*Micaelamys namaquensis*	11	0	10	0
	*Mus (Nannomys) minutoides*	29	0	12	0
	*Rattus* sp.	2	0	0	0
	*Saccostomus campestris*	19	0	19	0
	*Steatomys* sp.	13	0	1	0
	*Steatomys pratensis*	21	0	16	0
Total amount of samples tested for Meletse		262	0	211	36
**Secunda, Mpumalanga, South Africa (*n* = 102)**					
Shrews (*n* = 3)	*Crocidura* sp.	2	0	0	0
	*Suncus* sp.	1	0	0	0
Rodents (*n* = 101)	*Mastomys* sp.	40	0	26	0
	*Mastomys natalensis*	3	0	3	0
	*Rhabdomys* sp.	21	0	6	0
Total amount of samples tested for Secunda		67	0	35	0

Note: Positive results are indicated in bold.

Sp., species.

**TABLE 2-A1:** Sampling event details of collected *Gerbilliscus leucogaster* serum samples from Meletse, Limpopo province and their seropositivity.

Sampling (Month & year)	Associated season†	Number of serum samples collected	Number of serum samples positive for MOKV VNAs	Percentage	Seropositivity
February 2015	Summer	1	1	100.00	1/1
January 2016	Summer	1	0	0.00	0/1
June 2016	Winter	1	1	100.00	1/1
September 2016	Spring	1	1	100.00	1/1
November 2016	Spring	1	1	100.00	1/1
March 2017	Autumn/Fall	17	15	88.24	15/17
May 2017	Autumn/Fall	9	7	77.78	7/9
August 2017	Winter	3	3	100.00	3/3
November 2017	Spring	7	7	100.00	100/100

**Total**		**41**	**36**	**87.80%**	**36/41**

MOKV VNAs, Mokola virus neutralising antibodies.

† , Season delineation in South Africa: (1) Summer, 01 December to 28/29 February; (2) Autumn/Fall, 01 March to 31 May; (3) Winter, 01 June to 31 August; (4) Spring, 01 September to 30 November.

**TABLE 3-A1:** The 50% end-point neutralisation titres of all *Gerbilliscus leucogaster* serum samples from Meletse, Limpopo province that were positive for the presence of Mokola virus neutralising antibodies.

Reference number	Serum sample information		Initial screening‡				Follow-up screening‡										MOKV VNA titre
	Sample collection date	Sex	1:10 (i)	1:25 (i)	1:10 (f)	1:25 (f)	1:10 (i)	1:50 (i)	1:250 (i)	1:1250 (i)	Log ED_50_§ (i)	1:10 (f)	1:50 (f)	1:250 (f)	1:1250 (f)	Log ED_50_§ (f)	Average Log ED_50_ ± s.d.¶
UP4962†	26 Feb 2015	M	1	3	1	3	1	6	9	10	1.59	1	6	10	10	1.53	1.56 ± 0.03
UP12086†	08 June 2016	M	0	2	0	2	0	5	8	10	1.83	1	5	8	10	1.76	1.80 ± 0.04
UP12133†	06 Sept. 2016	M	2	4	2	4	2	6	9	10	1.53	2	6	10	10	1.48	1.51 ± 0.03
UP12166†	09 Nov. 2016	M	0	1	0	2	0	3	6	9	2.17	0	3	7	10	2.05	2.11 ± 0.06
UP12183	27 Mar. 2017	F	1	2	0	2	0	4	8	10	1.92	0	5	9	10	1.77	1.85 ± 0.08
UP12185†	27 Mar. 2017	F	0	3	1	3	0	4	8	9	1.97	0	4	8	10	1.92	1.95 ± 0.03
UP12187†	27 Mar. 2017	F	1	5	1	4	0	6	8	10	1.40	1	6	9	10	1.58	1.49 ± 0.09
UP12193†	28 Mar. 2017	M	0	1	0	1	0	3	7	9	2.10	0	4	8	10	1.92	2.01 ± 0.09
UP12194†	28 Mar. 2017	F	0	0	0	0	0	3	7	10	2.05	0	3	7	10	2.05	2.05
UP12195†	28 Mar. 2017	M	0	0	1	0	0	2	8	10	2.05	0	3	7	10	2.05	2.05
UP12196††	28 Mar. 2017	F	1	0	0	0	-	-	-	-	-	-	-	-	-	-	-
UP12197†	28 Mar. 2017	M	0	0	0	0	0	5	8	10	1.83	0	4	9	10	1.87	1.85 ± 0.02
UP12202†	28 Mar. 2017	F	1	3	0	4	0	6	8	10	1.40	0	6	8	10	1.40	1.40
UP12207†	28 Mar. 2017	M	0	0	0	0	0	1	5	8	2.46	0	2	4	8	2.51	2.49 ± 0.02
UP12208†	28 Mar. 2017	F	0	2	0	1	0	7	10	10	1.50	0	8	10	10	1.44	1.47 ± 0.03
UP12221	29 Mar. 2017	F	0	2	0	1	0	6	9	10	1.64	0	7	10	10	1.50	1.57 ± 0.07
UP12223†	29 Mar. 2017	M	1	3	0	2	0	4	7	10	1.98	1	5	8	10	1.76	1.87 ± 0.11
UP12246†	30 Mar. 2017	M	0	5	1	6	0	8	10	10	1.44	0	8	10	10	1.44	1.44
UP12259†	31 Mar. 2017	F	0	5	0	4	0	8	10	10	1.44	0	8	10	10	1.44	1.44
UP12296†	16-May-2017	M	0	4	1	5	1	7	10	10	1.45	2	8	10	10	1.35	1.40 ± 0.05
UP12297†	16 May 2017	F	1	5	0	5	0	7	10	10	1.50	0	7	10	10	1.50	1.50
UP12303†	16 May 2017	F	2	4	2	5	1	7	10	10	1.45	2	8	10	10	1.35	1.40 ± 0.05
UP12307†	16 May 2017	F	0	0	0	0	0	3	6	9	2.17	0	4	6	10	2.05	2.11 ± 0.06
UP12350	18 May 2017	M	2	5	1	5	1	7	10	10	1.45	1	8	10	10	1.40	1.42 ± 0.03
UP12354	18 May 2017	F	0	3	0	2	0	5	8	10	1.83	0	5	9	10	1.77	1.80 ± 0.03

UP, University of Pretoria; MOKV VNA, Mokola virus neutralising antibodies ; ED_50_, effective dose; s.d., standard deviation; F, female; M, male.

† , Small non-volant mammal individuals that were collected as voucher specimens and whose brains were negative for the presence of MOKV RNA. Animal ethics clearance was obtained from the University of Pretoria’s Animal Ethics Committee (Reference Numbers: EC071-15 & H008-18);

‡ , Results for the 1:10, 1:25, 1:50, 1:250 & 1:1250 serum dilutions are recorded as a number that represents the number of fields (out of a total of 10) that contain MOKV 12/458 infected cells for both initial (i) and duplicate (f) rounds of the micro-neutralisation test;

§ , The log10 50% end-point (ED) neutralisation titre for each serum sample as calculated by Reed and Muench (1938);

¶ , The average log10 50% ED neutralisation titre, together with the standard deviation (s.d.) for each serum sample as calculated by Reed and Muench (1938);

†† , Follow-up screening could not be completed as the serum sample was depleted during initial screening.

**TABLE 4-A1:** Micro-neutralisation test results of all non-volant small mammal serum samples that tested negative for the presence of Mokola virus neutralising antibodies.

Reference number	Serum sample information			Initial screening†			
	Sample collection date	Non-volant small mammal type	Non-volant small mammal species	1:10 (i)	1:25 (i)	1:10 (f)	1:25 (f)
**Meletse, Limpopo, South Africa (*n* = 211)**							
4961	26 Feb. 2015	Rodent	*Aethomys chrysophilus*	10	10	9	10
4963	26 Feb. 2015	Rodent	*Aethomys chrysophilus*	10	10	10	10
4967	27 Feb. 2015	Rodent	*Aethomys chrysophilus*	10	10	10	10
4968	27 Feb. 2015	Rodent	*Saccostomus campestris*	7	10	8	10
4969	27 Feb. 2015	Rodent	*Aethomys chrysophilus*	7	10	8	10
5011	03 Mar. 2015	Rodent	*Steatomys pratensis*	10	10	10	10
5012	03 Mar. 2015	Rodent	*Aethomys chrysophilus*	7	9	7	10
5015	04 Mar. 2015	Rodent	*Steatomys pratensis*	9	10	10	10
5017	05 Mar. 2015	Rodent	*Steatomys pratensis*	8	10	10	10
5352	12 May 2015	Rodent	*Aethomys ineptus*	10	10	9	10
5353	12 May 2015	Sengi	*Elephantulus brachyrhynchus*	10	10	10	10
5354	12 May 2015	Rodent	*Mastomys coucha*	10	10	10	10
5355	12 May 2015	Sengi	*Elephantulus brachyrhynchus*	9	10	10	10
5356	12 May 2015	Rodent	*Mastomys natalensis*	10	9	10	10
5525	22 July 2015	Rodent	*Aethomys chrysophilus*	8	10	9	10
5527	23 July 2015	Shrew	*Crocidura maquassiensis*	10	10	10	10
5528	24 July 2015	Rodent	*Mastomys coucha*	10	10	9	10
5529	24 July 2015	Rodent	*Mus (Nannomys) minutoides*	9	10	10	10
5934	15 Sept. 2015	Rodent	*Mus (Nannomys) minutoides*	9	9	10	10
5935	15 Sept. 2015	Rodent	*Micaelamys namaquensis*	9	10	10	10
5936	15 Sept. 2015	Rodent	*Aethomys ineptus*	10	10	9	10
5939	17 Sept. 2015	Rodent	*Mus (Nannomys) minutoides*	9	10	8	10
12001	10 Nov. 2015	Rodent	*Mastomys coucha*	10	10	10	10
12003	11 Nov. 2015	Rodent	*Aethomys chrysophilus*	10	10	10	10
12004	11 Nov. 2015	Rodent	*Steatomys pratensis*	10	10	10	10
12005	12 Nov. 2015	Rodent	*Aethomys ineptus*	5	9	6	10
12006	13 Nov. 2015	Rodent	*Aethomys ineptus*	10	10	10	10
12007	13 Nov. 2015	Rodent	*Steatomys pratensis*	10	10	10	10
12010	19 Jan. 2016	Rodent	*Mastomys coucha*	10	10	10	10
12011	19 Jan. 2016	Rodent	*Steatomys pratensis*	10	10	10	10
12012	19 Jan. 2016	Rodent	*Gerbilliscus leucogaster*	10	10	10	10
12018	20 Jan. 2016	Rodent	*Mastomys coucha*	10	10	10	10
12019	20 Jan. 2016	Rodent	*Saccostomus campestris*	9	10	10	10
12020	20 Jan. 2016	Rodent	*Mastomys coucha*	9	10	10	10
12023	20 Jan. 2016	Rodent	*Saccostomus campestris*	10	10	9	10
12062	05 Apr. 2016	Rodent	*Saccostomus campestris*	10	9	10	10
12065	06 Apr. 2016	Rodent	*Saccostomus campestris*	10	10	10	10
12066	06 Apr. 2016	Rodent	*Graphiurus murinus*	10	10	10	10
12067	06 Apr. 2016	Rodent	*Aethomys ineptus*	9	10	10	10
12075	07 June 2016	Rodent	*Mastomys natalensis*	10	10	9	10
12081	07 June 2016	Rodent	*Aethomys ineptus*	2	8	2	8
12082	07 June 2016	Rodent	*Aethomys ineptus*	10	10	10	10
12083	07 June 2016	Rodent	*Acomys selousi*	10	10	10	10
12084	08 June 2016	Rodent	*Saccostomus campestris*	8	10	9	10
12085	08 June 2016	Rodent	*Micaelamys namaquensis*	10	10	10	10
12087	08 June 2016	Rodent	*Aethomys ineptus*	10	10	9	10
12088	09 June 2016	Rodent	*Aethomys chrysophilus*	10	10	10	10
12132	06 Sept. 2016	Rodent	*Aethomys ineptus*	9	9	10	10
12134	06 Sept. 2016	Rodent	*Micaelamys sp.*	8	10	10	10
12140	07 Sept. 2016	Rodent	*Micaelamys sp.*	9	9	10	10
12142	07 Sept. 2016	Sengi	*Elephantulus brachyrhynchus*	9	9	10	10
12143	08 Sept. 2016	Rodent	*Micaelamys sp.*	8	9	10	10
12145	09 Sept. 2016	Rodent	*Micaelamys namaquensis*	9	10	9	10
12146	09 Sept. 2016	Rodent	*Acomys selousi*	10	10	9	10
12147	09 Sept. 2016	Rodent	*Steatomys pratensis*	10	10	9	10
12148	09 Sept. 2016	Rodent	*Saccostomus campestris*	10	10	10	10
12149	09 Sept. 2016	Rodent	*Saccostomus campestris*	10	10	10	10
12154	08 Nov. 2016	Rodent	*Micaelamys namaquensis*	9	10	10	10
12157	08 Nov. 2016	Rodent	*Aethomys chrysophilus*	9	10	10	10
12159	09 Nov. 2016	Rodent	*Saccostomus campestris*	9	10	10	10
12167	09 Nov. 2016	Rodent	*Aethomys chrysophilus*	9	9	10	10
12168	10 Nov. 2016	Rodent	*Micaelamys namaquensis*	8	9	10	10
12169	10 Nov. 2016	Rodent	*Mus (Nannomys) minutoides*	10	10	10	10
12170	10 Nov. 2016	Rodent	*Aethomys chrysophilus*	8	10	7	10
12171	11 Nov. 2016	Rodent	*Acomys spp.*	9	10	8	10
12176	07 Feb. 2017	Rodent	*Saccostomus campestris*	3	9	3	10
12177	08 Feb. 2017	Rodent	*Aethomys chrysophilus*	9	10	7	10
12178	08 Feb. 2017	Rodent	*Aethomys ineptus*	6	9	7	9
12179	08 Feb. 2017	Rodent	*Aethomys ineptus*	6	9	7	9
12180	09 Feb. 2017	Rodent	*Aethomys chrysophilus*	10	10	9	10
12181	09 Feb. 2017	Rodent	*Aethomys chrysophilus*	10	10	10	10
12184	27 Mar. 2017	Rodent	*Gerbilliscus leucogaster*	3	6	2	6
12188	27 Mar. 2017	Rodent	*Saccostomus campestris*	9	9	10	10
12189	27 Mar. 2017	Rodent	*Saccostomus campestris*	7	10	6	9
12190	27 Mar. 2017	Rodent	*Steatomys pratensis*	10	10	10	10
12191	27 Mar. 2017	Rodent	*Steatomys pratensis*	10	10	10	10
12192	28 Mar. 2017	Rodent	*Steatomys pratensis*	10	10	9	10
12200	28 Mar. 2017	Rodent	*Gerbilliscus leucogaster*	4	7	5	7
12201	28 Mar. 2017	Rodent	*Steatomys pratensis*	10	10	9	10
12203	28 Mar. 2017	Rodent	*Mastomys coucha*	6	9	6	10
12204	28 Mar. 2017	Rodent	*Aethomys ineptus*	7	10	8	10
12205	28 Mar. 2017	Rodent	*Mastomys coucha*	9	10	10	10
12206	28 Mar. 2017	Rodent	*Mastomys coucha*	5	9	8	9
12209	29 Mar. 2017	Rodent	*Aethomys chrysophilus*	8	10	7	10
12210	29 Mar. 2017	Rodent	*Mastomys coucha*	10	10	10	10
12211	29 Mar. 2017	Rodent	*Steatomys pratensis*	9	10	8	10
12212	29 Mar. 2017	Rodent	*Aethomys chrysophilus*	8	10	9	10
12213	29 Mar. 2017	Shrew	*Crocidura hirta*	9	10	8	9
12215	29 Mar. 2017	Rodent	*Mus (Nannomys) minutoides*	7	10	8	9
12216	29 Mar. 2017	Rodent	*Steatomys pratensis*	10	10	10	10
12217	29 Mar. 2017	Rodent	*Mastomys coucha*	8	10	7	10
12218	29 Mar. 2017	Rodent	*Saccostomus campestris*	8	9	10	10
12219	29 Mar. 2017	Rodent	*Steatomys pratensis*	8	10	8	10
12236	30 Mar. 2017	Rodent	*Saccostomus campestris*	9	10	8	10
12237	30 Mar. 2017	Rodent	*Mus (Nannomys) minutoides*	9	10	9	10
12238	30 Mar. 2017	Rodent	*Mastomys coucha*	7	10	6	10
12240	30 Mar. 2017	Rodent	*Saccostomus campestris*	10	10	10	10
12242	30 Mar. 2017	Rodent	*Aethomys ineptus*	9	10	7	10
12244	30 Mar. 2017	Rodent	*Saccostomus campestris*	10	10	10	10
12248	30 Mar. 2017	Rodent	*Saccostomus campestris*	10	10	10	10
12256	31 Mar. 2017	Rodent	*Graphiurus murinus*	8	10	10	10
12258	31 Mar. 2017	Rodent	*Steatomys pratensis*	10	10	10	10
12260	31 Mar. 2017	Rodent	*Steatomys pratensis*	10	10	10	10
12285	16 May 2017	Rodent	*Micaelamys namaquensis*	8	9	9	10
12289	16 May 2017	Rodent	*Saccostomus campestris*	10	10	10	10
12291	16 May 2017	Rodent	*Micaelamys namaquensis*	9	10	10	10
12292	16 May 2017	Rodent	*Aethomys ineptus*	9	10	10	10
12294	16 May 2017	Rodent	*Aethomys ineptus*	8	10	8	10
12295	16 May 2017	Rodent	*Aethomys ineptus*	10	10	10	10
12298	16 May 2017	Rodent	*Mastomys coucha*	10	10	10	10
12299	16 May 2017	Rodent	*Mus (Nannomys) minutoides*	10	10	10	10
12300	16 May 2017	Rodent	*Mastomys coucha*	9	10	10	10
12301	16 May 2017	Rodent	*Mastomys coucha*	10	10	10	10
12302	16 May 2017	Rodent	*Aethomys sp.*	10	10	10	10
12304	16 May 2017	Rodent	*Aethomys sp.*	10	10	10	10
12305	16 May 2017	Rodent	*Lemniscomys rosalia*	9	10	10	10
12306	16 May 2017	Rodent	*Mastomys coucha*	9	10	10	10
12311	17 May 2017	Rodent	*Mastomys natalensis*	9	10	10	10
12319	17 May 2017	Rodent	*Micaelamys namaquensis*	9	10	10	10
12320	17 May 2017	Rodent	*Gerbilliscus leucogaster*	1	6	2	7
12321	17 May 2017	Rodent	*Aethomys ineptus*	8	10	10	10
12324	17 May 2017	Rodent	*Gerbilliscus leucogaster*	9	10	10	10
12329	17 May 2017	Rodent	*Mastomys coucha*	10	10	10	10
12331	17 May 2017	Rodent	*Mastomys natalensis*	10	10	10	10
12332	17 May 2017	Rodent	*Mastomys coucha*	10	10	10	10
12348	18 May 2017	Rodent	*Mastomys coucha*	10	10	10	10
12349	18 May 2017	Rodent	*Aethomys sp.*	10	10	10	10
12356	18 May 2017	Rodent	*Aethomys sp.*	10	10	10	10
12365	18 May 2017	Rodent	*Aethomys sp.*	7	10	10	10
12366	18 May 2017	Rodent	*Mus (Nannomys) minutoides*	10	10	10	10
12367	18 May 2017	Rodent	*Mastomys coucha*	7	10	8	10
12374	18 May 2017	Rodent	*Aethomys ineptus*	9	10	10	10
12375	18 May 2017	Rodent	*Mastomys natalensis*	10	10	10	10
12376	18 May 2017	Rodent	*Mastomys coucha*	9	10	10	10
12377	19 May 2017	Rodent	*Mus (Nannomys) minutoides*	7	10	10	10
12378	18 May 2017	Rodent	*Steatomys sp.*	10	10	10	10
12380	19 May 2017	Rodent	*Aethomys ineptus*	10	10	10	10
12381	19 May 2017	Rodent	*Mastomys natalensis*	10	10	10	10
12394	29 Aug. 2017	Rodent	*Mastomys natalensis*	6	10	5	10
12395	29 Aug. 2017	Rodent	*Mastomys natalensis*	10	10	10	10
12405	29 Aug. 2017	Rodent	*Aethomys sp.*	10	10	10	10
12406	29 Aug. 2017	Rodent	*Aethomys sp.*	10	10	10	10
12409	29 Aug. 2017	Rodent	*Aethomys sp.*	10	10	9	10
12414	29 Aug. 2017	Sengi	*Elephantulus brachyrhynchus*	10	10	10	10
12422	29 Aug. 2017	Rodent	*Aethomys sp.*	9	10	10	10
12423	29 Aug. 2017	Rodent	*Aethomys sp.*	9	10	9	10
12428	29 Aug. 2017	Rodent	*Lemniscomys rosalia*	10	10	10	10
12434	29 Aug. 2017	Rodent	*Aethomys ineptus*	10	10	10	10
12435	29 Aug. 2017	Rodent	*Aethomys ineptus*	10	10	10	10
12438	29 Aug. 2017	Rodent	*Aethomys sp.*	10	10	10	10
12442	29 Aug. 2017	Rodent	*Aethomys sp.*	9	10	10	10
12443	29 Aug. 2017	Rodent	*Lemniscomys rosalia*	9	10	10	10
12447	30 Aug. 2017	Rodent	*Micaelamys namaquensis*	10	10	10	10
12449	30 Aug. 2017	Rodent	*Aethomys sp.*	10	10	10	10
12452	30 Aug. 2017	Rodent	*Aethomys sp.*	9	10	9	10
12453	30 Aug. 2017	Rodent	*Aethomys sp.*	10	10	10	10
12458	30 Aug. 2017	Rodent	*Mastomys coucha*	10	10	10	10
12468	30 Aug. 2017	Rodent	*Aethomys sp.*	10	10	10	10
12474	31 Aug. 2017	Rodent	*Aethomys ineptus*	10	10	10	10
12475	31 Aug. 2017	Rodent	*Lemniscomys rosalia*	10	10	10	10
12515	21 Nov. 2017	Rodent	*Mastomys natalensis*	10	10	10	10
12516	21 Nov. 2017	Rodent	*Lemniscomys rosalia*	10	10	10	10
12519	21 Nov. 2017	Rodent	*Mastomys natalensis*	10	10	10	10
12520	21 Nov. 2017	Rodent	*Lemniscomys rosalia*	7	10	10	10
12521	21 Nov. 2017	Rodent	*Mastomys natalensis*	10	10	10	10
12525	21 Nov. 2017	Rodent	*Mastomys natalensis*	10	10	10	10
12538	21 Nov. 2017	Rodent	*Lemniscomys rosalia*	8	10	7	10
12547	23 Nov. 2017	Shrew	*Crocidura hirta*	10	10	10	10
12549	23 Nov. 2017	Rodent	*Mus (Nannomys) minutoides*	9	10	10	10
12552	23 Nov. 2017	Rodent	*Aethomys ineptus*	10	10	10	10
12556	23 Nov. 2017	Rodent	*Micaelamys namaquensis*	10	10	10	10
12557	23 Nov. 2017	Rodent	*Mus (Nannomys) minutoides*	7	10	6	10
12558	23 Nov. 2017	Rodent	*Saccostomus campestris*	6	10	6	10
12562	24 Nov. 2017	Rodent	*Mus (Nannomys) minutoides*	10	10	10	10
12563	24 Nov. 2017	Rodent	*Mastomys coucha*	10	10	10	10
**Secunda, Mpumalanga, South Africa (*n* = 35)**							
5532	30 June 2015	Rodent	*Mastomys* sp.	9	9	9	10
5552	01 July 2015	Rodent	*Mastomys* sp.	10	10	10	10
5553	01 July 2015	Rodent	*Mastomys* sp.	4	6	5	6
5566	02 July 2015	Rodent	*Mastomys* sp.	9	10	10	9
5567	02 July 2015	Rodent	*Mastomys* sp.	9	10	10	10
5568	02 July 2015	Rodent	*Rhabdomys* sp.	10	10	10	10
5569	02 July 2015	Rodent	*Rhabdomys* sp.	10	10	10	10
5570	03 July 2015	Rodent	*Mastomys* sp.	10	10	10	9
5571	03 July 2015	Rodent	*Mastomys* sp.	10	9	10	10
5572	03 July 2015	Rodent	*Rhabdomys* sp.	10	10	10	10
5573	03 July 2015	Rodent	*Mastomys* sp.	10	10	10	10
5574	03 July 2015	Rodent	*Mastomys* sp.	10	10	10	10
5575	03 July 2015	Rodent	*Rhabdomys* sp.	9	10	10	9
5576	03 July 2015	Rodent	*Rhabdomys* sp.	9	10	10	10
5577	03 July 2015	Rodent	*Mastomys* sp.	9	10	10	10
5580	03 July 2015	Rodent	*Mastomys* sp.	10	10	10	10
5581	03 July 2015	Rodent	*Mastomys* sp.	9	10	9	9
5582	03 July 2015	Rodent	*Mastomys* sp.	10	10	9	10
5584	03 July 2015	Rodent	*Mastomys* sp.	10	10	10	10
5585	03 July 2015	Rodent	*Mastomys* sp.	9	9	10	9
5586	03 July 2015	Rodent	*Mastomys* sp.	9	10	10	10
12025	26 Jan. 2016	Rodent	Mastomys natalensis	9	10	9	10
12026	26 Jan. 2016	Rodent	Mastomys natalensis	10	10	10	10
12027	26 Jan. 2016	Rodent	Mastomys natalensis	10	10	10	10
12028	27 Jan. 2016	Rodent	*Mastomys* sp.	9	10	8	10
12029	27 Jan. 2016	Rodent	*Mastomys* sp.	10	10	10	10
12030	27 Jan. 2016	Rodent	*Mastomys* sp.	10	10	10	10
12031	27 Jan. 2016	Rodent	*Rhabdomys* sp.	10	10	10	10
12032	27 Jan. 2016	Rodent	*Mastomys* sp.	5	8	5	7
12034	27 Jan. 2016	Rodent	*Mastomys* sp.	10	10	10	10
12035	28 Jan. 2016	Rodent	*Mastomys* sp.	8	10	3	10
12036	28 Jan. 2016	Rodent	*Mastomys* sp.	10	10	10	10
12037	28 Jan. 2016	Rodent	*Mastomys* sp.	10	10	10	10
12038	28 Jan. 2016	Rodent	*Mastomys* sp.	9	10	10	10
12050	29 Jan. 2016	Rodent	*Mastomys* sp.	10	10	10	10

Sp., species.

† , Results for the 1:10 and 1:25 serum dilutions are recorded as a number that represents the number of fields (out of a total of 10) that contain MOKV 12/458 infected cells for both initial (i) and duplicate (f) rounds of the micro-neutralisation test.

## References

[CIT0001] Aghomo, H.O., Tomori, O., Oduye, O.O. & Rupprecht, C.E., 1990, ‘Detection of Mokola virus neutralising antibodies in Nigerian dogs’, *Research in Veterinary Science* 48(2), 264. 10.1016/S0034-5288(18)31005-1 2333438

[CIT0002] Aghová, T., Šumbera, R., Piálek, L., Mikula, O., McDonough, M.M., Lavrenchenko, L.A. et al., 2017, ‘Multilocus phylogeny of East African gerbils (Rodentia, Gerbilliscus) illuminates the history of the Somali-Masai savanna’, *Journal of Biogeography* 44(10), 2295–2307. 10.1111/jbi.13017

[CIT0003] Causey, O.R., Kemp, G.E., Madbouly, M.H. & Lee, V.H., 1969, ‘Arbovirus surveillance in Nigeria, 1964–1967’, *Bulletin de la Société de Pathologie Exotique* 62(2), 249–253. 5409104

[CIT0004] Choate, T.S., 1972, ‘Behavioural studies on some Rhodesian rodents’, *African Zoology* 7(1), 103–118. 10.1080/00445096.1972.11447433

[CIT0005] Coertse, J., Markotter, W., Le Roux, K., Stewart, D., Sabeta, C.T. & Nel, L.H., 2017, ‘New isolations of the rabies-related Mokola virus from South Africa’, *BMC Veterinary Research* 13(1), 37. 10.1186/s12917-017-0948-0 28143485 PMC5282659

[CIT0006] Coertse, J., Weyer, J., Nel, L.H. & Markotter, W., 2019, ‘Reverse transcription recombinase polymerase amplification assay for rapid detection of canine associated rabies virus in Africa’, *PLoS One* 14(7), e0219292. 10.1371/journal.pone.0219292 31276479 PMC6611627

[CIT0007] Colangelo, P., Granjon, L., Taylor, P.J. & Corti, M., 2007, ‘Evolutionary systematics in African gerbilline rodents of the genus Gerbilliscus: Inference from mitochondrial genes’, *Molecular Phylogenetics and Evolution* 42(3), 797–806. 10.1016/j.ympev.2006.10.001 17113792

[CIT0008] De Graaff, G., 1981, *The rodents of southern Africa: Notes on their identification, distribution, ecology, and taxonomy*, Butterworth-Heinemann, Oxford, United Kingdom.

[CIT0009] Dempster, E.R., 2013, ‘Gerbilliscus leucogaster’, *Mammals of Africa* 3, 279–281.

[CIT0010] Foggin, C.M., 1988, ‘Rabies and rabies-related viruses in Zimbabwe: Historical, virological and ecological aspects’, PhD thesis, University of Zimbabwe, Harare.

[CIT0011] Granjon, L. & Dempster, E.R., 2013, ‘Genus Gerbilliscus gerbils’, *Mammals of Africa* 3, 268–270.

[CIT0012] Greenberg, J.A., DiMenna, M.A., Hanelt, B. & Hofkin, B.V., 2012, ‘Analysis of post-blood meal flight distances in mosquitoes utilizing zoo animal blood meals’, *Journal of Vector Ecology* 37(1), 83–89. 10.1111/j.1948-7134.2012.00203.x 22548540 PMC3342775

[CIT0013] Kemp, G.E., Causey, O.R., Moore, D.L., Odelola, A. & Fabiyi, A., 1972, ‘Mokola virus’, *The American Journal of Tropical Medicine and Hygiene* 21(3), 356–359. 10.4269/ajtmh.1972.21.356 5025622

[CIT0014] Kgaladi, J., Wright, N., Coertse, J., Markotter, W., Marston, D., Fooks, A.R. et al., 2013, ‘Diversity and epidemiology of Mokola virus’, *PLoS Neglected Tropical Diseases* 7(10), e2511. 10.1371/journal.pntd.0002511 24205423 PMC3812115

[CIT0015] Kuzmin, I.V., Niezgoda, M., Franka, R., Agwanda, B., Markotter, W., Beagley, J.C. et al., 2008, ‘Lagos bat virus in Kenya’, *Journal of Clinical Microbiology* 46(4), 1451–1461. 10.1128/JCM.00016-08 18305130 PMC2292963

[CIT0016] Le Gonidec, G., Rickenbach, A., Robin, Y. & Heme, G., 1978, ‘Isolement d’une souche de virus Mokola au Cameroun’, *Annales des Microbiologie (Institute Pasteur)* 129(A), 245–249. 677617

[CIT0017] Meester, J.A.J., Rautenbach, I.L., Dippenaar, N.J. & Baker, C.M., 1986, ‘Classification of Southern African mammals’, *Transvaal Museum Monograph* 5(1), 1–359.

[CIT0018] Monadjem, A., Taylor, P.J., Denys, C. & Cotterill, F.P., 2015, *Rodents of sub-Saharan Africa: A biogeographic and taxonomic synthesis*, Walter de Gruyter GmbH & Co. KG, Berlin, Germany

[CIT0019] Newbery, C.H., 1999, ‘A key to the Soricidae, Macroscelididae, Gliridae and Muridae of Gauteng, North West Province, Mpumalanga and the Northern province, South Africa’, *Koedoe* 42(1), 51–55. 10.4102/koedoe.v42i1.221

[CIT0020] Nottidge, H.O., Omobowale, T.O. & Oladiran, O.O., 2007, ‘Mokola virus antibodies in humans, dogs, cats, cattle, sheep, and goats in Nigeria’, *International Journal of Applied Research in Veterinary Medicine* 5(3), 105.

[CIT0021] Ogunkoya, A.B., Beran, G.W., Umoh, J.U., Gomwalk, N.E. & Abdulkadir, I.A., 1990, ‘Serological evidence of infection of dogs and man in Nigeria by lyssaviruses (family *Rhabdoviridae*)’, *Transactions of the Royal Society of Tropical Medicine and Hygiene* 84(6), 842–845. 10.1016/0035-9203(90)90103-L 2096520

[CIT0022] Rautenbach, I.L., 1982, *Mammals of the Transvaal*, Ecoplan monograph no. 1:1–211, Transvaal Museum, Pretoria.

[CIT0023] Reed, L.J. & Muench, H., 1938, ‘A simple method of estimating fifty per cent endpoints’, *American Journal of Epidemiology* 27(3), 493–497. 10.1093/oxfordjournals.aje.a118408

[CIT0024] Saluzzo, J.F., Rollin, P.E., Dauguet, C., Digoutte, J.P., Georges, A.J. & Sureau, P., 1984, ‘Premier isolement du virus Mokola à partir d’un rongeur (*Lophuromys sikapusi*)’, *Annales de l’Institut Pasteur/Virologie* 135(1), 57–66. 10.1016/S0769-2617(84)80039-8

[CIT0025] Sikes, R.S. & Gannon, W.L., 2011, ‘Guidelines of the American society of mammalogists for the use of wild mammals in research’, *Journal of Mammalogy* 92(1), 235–253. 10.1644/10-MAMM-F-355.1 PMC5909806 29692469

[CIT0026] Skinner, J.D. & Chimimba, C.T., 2005, *The mammals of the Southern African sub-region*, Cambridge University Press, United Kingdom.

[CIT0027] Smithers, R.H.N., 1971, *A checklist of the mammals of Botswana*, Trustees of the National Museum of Rhodesia, Salisbury.

[CIT0028] Smith, T.G. & Gilbert, A.T., 2017, ‘Comparison of a micro-neutralization test with the rapid fluorescent focus inhibition test for measuring rabies virus neutralizing antibodies’, *Tropical Medicine and Infectious Disease* 2(3), 24. 10.3390/tropicalmed2030024 28845465 PMC5568636

[CIT0029] Walker, P.J., Breyta, R., Blasdell, K.R., Calisher, C.H., Dietzgen, R.G., Fooks, A.R. et al., 2018, ‘Rhabdoviridaee’, in J.H. Kuhn & S.G. Siddel (eds.), ICTV Report Negative-sense RNA viruses, *Journal of Gen eral Virology*, 99, 447–448. viewed 24 May 2020, from https://talk.ictvonline.org/ictv-reports/ictv_online_report/negative-sense-rna-viruses/w/rhabdoviridae

